# Year in review 2013: *critical* care - respiratory infections

**DOI:** 10.1186/s13054-014-0572-3

**Published:** 2014-10-29

**Authors:** Girish B Nair, Michael S Niederman

**Affiliations:** Pulmonary and Critical Care Medicine, Winthrop-University Hospital, 222 Station Plaza North, Suite 400, Mineola, NY 11501 USA; Department of Medicine, Winthrop-University Hospital, 222 Station Plaza North, Suite 509, Mineola, NY 11501 USA; Department of Medicine, SUNY at Stony Brook, 101 Nicolls Road, Stony Brook, NY 11794 USA

## Abstract

Infectious complications, particularly in the respiratory tract of critically ill patients, are related to increased mortality. Severe infection is part of a multiple system illness and female patients with severe sepsis have a worse prognosis compared to males. Kallistatin is a protective hormokine released during monocyte activation and low levels in the setting of septic shock can predict adverse outcomes. Presepsin is another biomarker that was recently evaluated and is elevated in patients with severe sepsis patients at risk of dying. The Centers for Disease Control and Prevention has introduced new definitions for identifying patients at risk of ventilator-associated complications (VACs), but several other conditions, such as pulmonary edema and acute respiratory distress syndrome, may cause VACs, and not all patients with VACs may have ventilator-associated pneumonia. New studies have suggested strategies to identify patients at risk for resistant pathogen infection and therapies that optimize efficacy, without the overuse of broad-spectrum therapy in patients with healthcare-associated pneumonia. Innovative strategies using optimized dosing of antimicrobials, maximizing the pharmacokinetic and pharmacodynamic properties of drugs in critically ill patients, and newer routes of drug delivery are being explored to combat drug-resistant pathogens. We summarize the major clinical studies on respiratory infections in critically ill patients published in 2013.

## Introduction

Critically ill patients with respiratory infections have been the continued focus of investigation over the last several years. Infections, mostly nosocomial, are a major cause of mortality in hospitalized patients related to an increased risk of infection with multi-drug resistant (MDR) pathogens and the widespread use of indiscriminate broad-spectrum antibiotics. The frequency and epidemiology of MDR pathogens show regional variation, however, with several studies pointing out that the risk of MDR pathogens in healthcare-associated pneumonia (HCAP) is variable and hence there is a need for accurate risk scoring in this category of patients. Obtaining meaningful data and monitoring trends of preventative strategies have become ever more important, with the Centers for Disease Control and Prevention (CDC) recently publishing new surveillance definitions. Newer biomarkers are becoming part of the increasing armamentarium in the field of critical care medicine and antibiotic stewardship using biomarkers has been studied robustly. Antibiotic use in the critically ill, with dosing to attain better pharmacokinetic and pharmacodynamic results, was part of several research studies. We summarize the findings from the major clinical research studies published in 2013 on respiratory infections, with a focus on infections in critically ill patients.

## Risk factors and outcomes

Respiratory infection continues to be the most common cause of sepsis and septic shock. The past decade has seen increased awareness in recognizing patients with sepsis and several guidelines, including the ‘Surviving Sepsis Campaign’, have published a detailed framework on the approach to patients with severe sepsis. In a large, prospective, French, multicenter, observational study as part of the EPISS study cohort, investigators examined the epidemiology of septic shock in 1,495 patients [1]. In this study, 53.6% of patients had respiratory tract infection as the cause of septic shock and 83.9% required invasive mechanical ventilation (MV), with Gram-negative bacilli being the most common pathogens identified. Although most patients received initial appropriate antibiotic therapy (n = 898), the in-hospital mortality rate was still high, up to 48.7%. A higher Sequential Organ Failure Assessment (SOFA) score, age and chronic health status score and the presence of immunosuppression were independent risk factors for short-term mortality. In a follow-up study of the same cohort of patients, 3-month mortality was 52.2%. Severity of illness, indicated by a higher SOFA score early after septic shock, impacted mortality the most, while co-morbid conditions such as cirrhosis, nosocomial infection and age influenced mortality after hospitalization [2].

In another prospective observational cohort of 1,000 patients with severe sepsis, Phua and colleagues studied the characteristics and outcomes of patients with a positive microbial culture (58.5%) compared with those whose culture was negative (41.5%) [3]. Respiratory infection was the most common cause of sepsis in both groups, and a lung source was determined as the primary cause of sepsis more often in patients with a negative culture than in patients with a positive culture (74.5% versus 59.9, *P* <0.001). Of all the pathogens identified, infection with *Pseudomonas aeruginosa* (PA) was associated with increased mortality (odds ratio (OR) 2.02, 95% confidence interval (CI) 1.08 to 3.79, *P* = 0.03). Patients with culture-negative sepsis had fewer comorbidities; these patients were more often women and had lower severity of illness than those with culture-positive sepsis. Although patients with positive culture had higher mortality, it was not an independent predictor of mortality on logistic regression analysis. Sakr and colleagues [4] studied the influence of gender on 3,902 patients with severe sepsis and found the frequency of severe sepsis and septic shock was lower in women than in men (6.0% versus 8.9%, *P* = 0.001) and the overall ICU mortality was not different in both sexes (20.1% versus 19.8%, *P* = 0.834). In the subset of patients with severe sepsis, however, female patients had worse survival than men (63.5% versus 46.4%, *P* = 0.007). Further studies on the impact of gender-specific hormonal and immunologic profile differences may uncover an explanation for these findings.

Cognitive dysfunction has been noted in patients following severe illness. In a study including 5,888 participants, the authors tested the hypothesis that a bidirectional relationship exists between pneumonia and dementia, with subclinical changes in cognition increasing the risk of pneumonia hospitalization and an accelerated decline in cognitive dysfunction occurring after pneumonia [5]. Three trajectories were identified longitudinally based on Teng’s modified mini mental state examination - no decline, minimal decline and severe decline. A low cognitive score prior to hospitalization increased the risk of pneumonia - a 10 point lower modified mini mental state score increased the hazard of pneumonia by 8.4%. Patients who had at least one episode of pneumonia had a higher risk of developing subsequent dementia than those without pneumonia (hazards ratio 2.24, 95% CI 1.62 to 3.11, *P* = 0.01). Of the total population, 6.8% had severe sepsis and similar cognitive decline as was seen with pneumonia. Neurotoxicity related to elevated cytokine levels and other co-morbid conditions with severe illness, such as delirium, could be a plausible explanation for the cognitive decline. However, the population in this study who developed pneumonia were slightly older and had abnormal scores on mini mental state examination and potentially were identified earlier in their course with longitudinal screening.

Enteral feeding is the desired mode of nutritional supplementation in critically ill patients, but patients receiving enteral nutrition may have gastroparesis and gastroesophageal reflux, putting them at risk for aspiration; therefore, measurement of gastric residual volume (GRV) is recommended in ventilated patients. Reignier and associates [6] in a randomized, non-inferiority, open-label, multicenter trial studied whether GRV monitoring every 6 hours and adjusting enteral feeding rates if the volume exceeded 250 ml would prevent ventilator-associated pneumonia (VAP). In this study, there was no difference in VAP incidence between patients who had GRV measured (n = 227) compared with the group (n = 222) who did not (16.7% versus 15.8%), and all the clinical outcomes, including mortality, were similar in both groups. Patients in whom GRV was not measured had a higher incidence of vomiting, but also a higher proportion of this group achieved the calorie target and had lower use of prokinetic agents. Although the study was done well, it was underpowered to determine the harmful effects related to vomiting and included mostly patients in the medical ICU and excluded patients with gastrointestinal bleeding. In a meta-analysis of 19 randomized controlled trials including 1,394 patients, Alhazzani and colleagues [7] reviewed the risk of pneumonia in patients receiving small bowel feeding compared to gastric feeding. Small bowel feeding was associated with reduced risk of pneumonia (relative risk 0.70, 95% CI 0.55 to 0.90, *P* = 0.004), but there was no difference in mortality, ventilator days or ICU length of stay (LOS) between the two groups. The study is limited, however, because the individual trials had small sample sizes, included severe pancreatitis patients and patients not in the ICU and used variable definitions of pneumonia. Insertion of the small bowel feeding tube can be technically difficult if done blindly and may need additional training with fluoroscopy and endoscopy procedures. Even though oropharyngeal bacterial translocation seems a likely cause of the development of VAP, it is unclear if monitoring gastric reserve volume or advancing the feeding tube to the small intestine clearly prevents VAP. Another identified risk factor for ventilator-associated respiratory infection (including VAP and ventilator-associated tracheobronchitis) is iatrogenic immune suppression (OR 3.34), a risk factor that has frequently been excluded in prior studies [8].

Shorr and colleagues [9] studied the factors leading to 30-day readmission in 977 culture-proven non-nosocomial pneumonia patients who survived to discharge following initial hospitalization to any of the nine participating hospitals in the same geographical area. The readmission rate was 19.3% (n = 149) within the 30-day period and was related to non-pneumonia causes such as chronic obstructive pulmonary disease (25%) and congestive heart failure (CHF) (22%). While pneumonia accounted for only 7.4% (n = 11) of readmissions, patients with HCAP were re-admitted more often than those with community-acquired pneumonia (CAP) (24.4% versus 4.1%, *P* <0.001) and had more comorbid conditions. The four independent variables associated with readmission on logistic regression analysis were long-term care admission prior to index hospitalization (OR = 2.15, *P* = 0.001), immunosuppressed state (OR = 1.93, *P* = 0.001), previous antibiotics (OR = 1.74, *P* = 0.009) and previous 90-day hospitalization (OR = 1.66, *P* = 0.014). These data suggest that readmission rates differ among groups of patients with pneumonia, and that patients with HCAP and those with baseline poor functional status have a higher likelihood of being readmitted than uncomplicated CAP patients.

## Role of biomarkers

Clinical algorithms based on biomarkers help with antibiotic de-escalation and possibly limit antibiotic over-exposure in patients with pneumonia, but their use in clinical practice has been variable. Procalcitonin (PCT), an inflammatory hormokine, is elevated in bacterial infection and helps with antibiotic stewardship, and risk stratification, particularly for respiratory infections. Presepsin (sCD14-ST) is another novel biomarker (soluble amino-terminal fragment of the cluster of differentiation (CD) marker protein CD14) in sepsis that is released into the circulation during monocyte activation. Kallistatin is an endogenous serine proteinase inhibitor that has a strong affinity to tissue kallikrein and is thought to have a protective role with a higher consumption in patients with severe sepsis.

**Figure 1:**
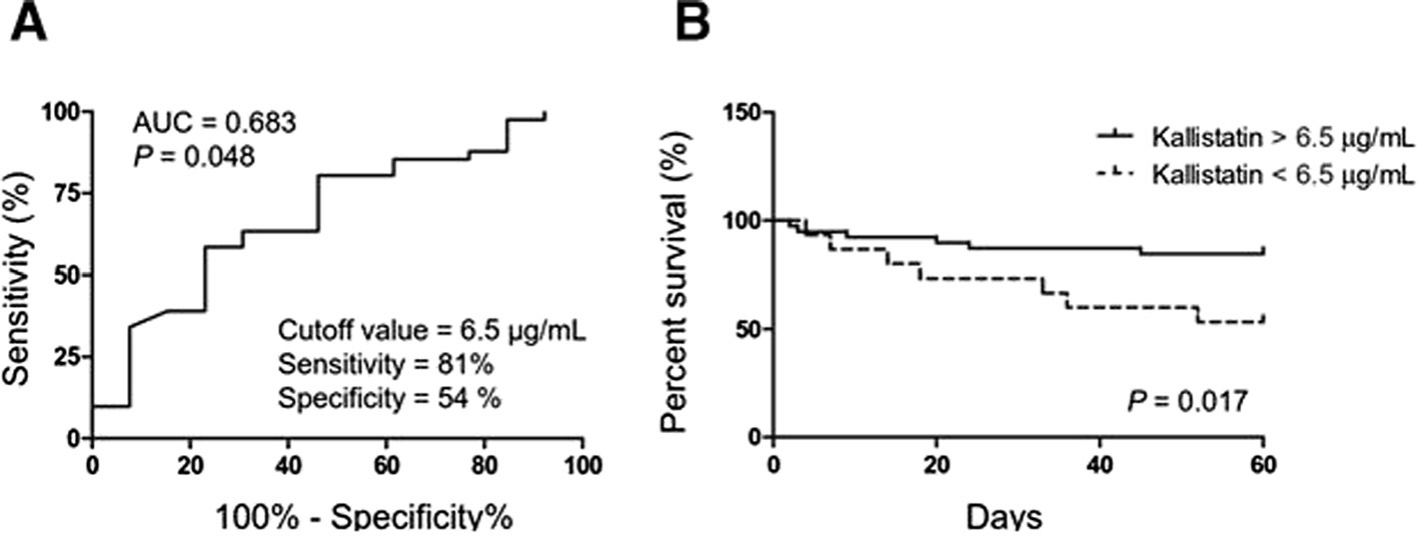
**Plasma kallistatin levels on day 1 of ICU admission and likelihood of 60-day survival. (A)** Receiver operating characteristic curves determining the cutoff value of day 1 kallistatin (6.5 μg/ml) to discriminate between survivors and nonsurvivors. **(B)** Kaplan-Meier curves of 60-day survival with patients grouped according to day 1 kallistatin levels >6.5 μg/ml or <6.5 μg/ml at ICU admission. Log-rank test was performed for comparisons between the groups. AUC, area under the curve. Adapted from Lin and colleagues [10].

In a prospective observational study of 54 severe CAP patients admitted to ICU, Lin and colleagues [10] determined the prognostic value of serum kallistatin and its correlation to other biomarkers; 17 healthy patients were included as controls. Plasma kallistatin and antithrombin III were significantly lower on days 1 and 4 in patients who did not survive (24%) compared to those who did, possibly indicating greater consumption of these factors in the severely ill. The plasma kallistatin levels were significantly reduced in patients with septic shock and in those who developed acute respiratory distress syndrome (ARDS). A cutoff level of day 1 kallistatin <6.5 μg/ml can discriminate between survivors and non-survivors with an area under the curve (AUC) of 0.683, *P* = 0.04 (Figure 1). Thus, decreased plasma kallistatin level on day 1 of ICU admittance is independently associated with mortality and severity of disease in CAP patients in this study. In a multicenter, case–control study, Masson and colleagues [11] compared presepsin and PCT levels in 50 survivors and 50 non-survivors who were admitted to ICU with severe sepsis. The presepsin levels were significantly higher on day 1 of enrollment in patients who died compared with survivors and remained significantly elevated on day 7 as well. Presepsin was independently associated with short-term ICU and 28-day mortality and had good prognostic accuracy similar to the SOFA score for long-term mortality at 90 days. PCT on the other hand was not related to mortality and the levels declined on day 7 in both survivors and non-survivors.

**Figure 2:**
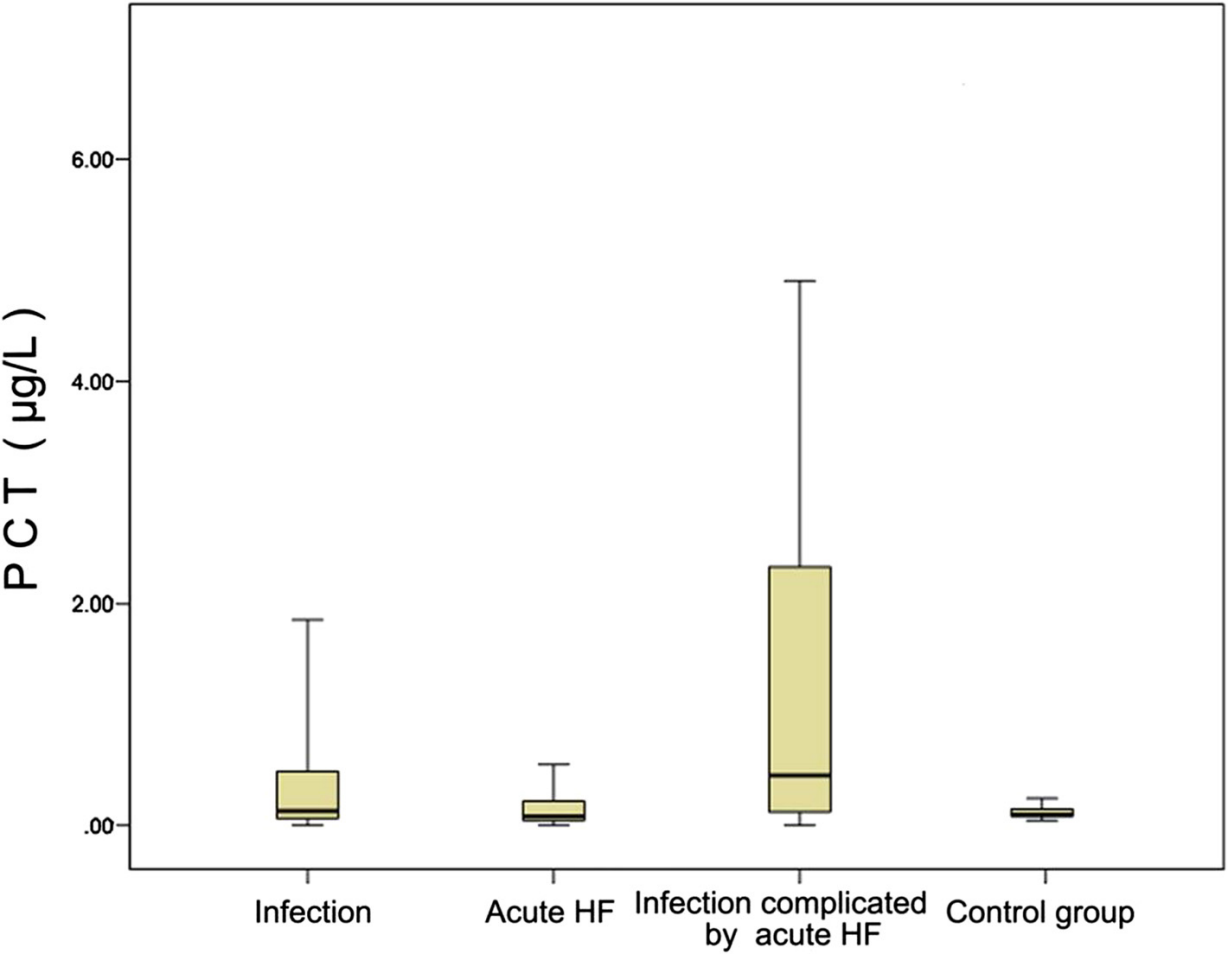
**Differential expression of procalcitonin in different populations.** Boxes represent interquartile range, and whiskers the 5th and 95th percentiles in each category. HF, heart failure; PCT, procalcitonin. Adapted from Wang and colleagues [12].

CHF may cause gut translocation of bacteria and potentially lead to elevated PCT levels. Wang and colleagues [12] studied the diagnostic value of serum PCT levels in 4,698 patients with different types of CHF. Patients were grouped into CHF (n = 1,364), CHF with infection (n = 1,183), infection only (n = 1,703) and healthy controls (n = 448). The PCT levels in patients with CHF were significantly elevated compared with healthy controls, while those with infection and CHF had higher levels than both the infection alone and CHF alone group (Figure 2). In patients with increasing severity of CHF, the positive predictive value of PCT decreased significantly (90.9 in class II CHF with infection to 68.6 in class IV CHF). If the PCT was negative, however, the finding was good for ruling out infection in class IV CHF patients (negative predictive value of 89). Hence, elevated PCT should not be taken at face value in patients with CHF and a higher cutoff should be used to define infection, depending on the severity of heart failure.

In a meta-analysis of seven studies including 1,075 patients, Prkno and colleagues [13] studied the safety of using a PCT-based regimen in patients with severe sepsis or septic shock. The 28-day mortality based on results from four included studies was not different between the PCT-based regimen and standard treatment groups, but the PCT group had a shorter duration of antimicrobial therapy based on five included studies. The studies included in the meta-analysis had substantial differences in design and cutoff values for PCT and included both medical and surgical patients, but the common theme was that therapy based on PCT leads to more de-escalation and a shorter duration of antibiotic therapy, with no adverse impact on mortality. The Stop Antibiotics on Guidance of Procalcitonin Study (SAPS) is an ongoing, multicenter, randomized Dutch study of daily PCT versus standard therapy , currently finishing enrollment, and will be the largest ICU-based trial evaluating the early stopping of antibiotics based on PCT [14].

## Surveillance strategies and antibiotic stewardship

Healthcare bundles in the form of daily goal sheets and educational sessions have been shown to reduce the incidence of VAP and related complications, but variable practices and different VAP definitions limit their use. The CDC recently introduced a step-wise approach for ‘objective’ surveillance of ventilator-associated events and includes ventilator-associated complications (VACs), infection-related ventilator-associated complications (IVACs), as well as possible and probable VAP.

Muscedere and associates [15] studied the clinical impact and preventability of VACs and IVACs using prospectively collected data on 1,320 patients from another series and determined the relationship to VAP. Over four study periods, VACs developed in 10.5% of patients (n = 139), IVACs in 4.9% (n = 65) and VAP was noted in 11.2% (n = 148); 39 patients had both a VAC or IVAC and VAP. Patients who had VACs were more likely to develop VAP than those who did not have VACs (28.1% versus 9.2%, *P* <0.001). Patients with VACs or IVACs had significantly more ventilator days, hospital days, and antibiotic days and higher hospital mortality compared with patients who did not develop VACs or IVACs. When prevention efforts were undertaken, they were able to reduce the incidence of VACs and VAP, but not IVACs, over subsequent periods. In another study, Hayashi and colleagues [16] compared 153 patients with VACs to 390 without VACs and noted that patients who developed VACs had a longer ICU LOS (22 versus 11 days), duration of MV (20 versus 5 days) and use of antibiotics but no difference in overall ICU mortality and hospital LOS. VAC definitions identified a ‘potential VAP’ (a VAC with positive culture of respiratory pathogens in respiratory specimens plus antibiotic prescription with intention to treat as VAP) in 30.7% of cases, but it was not specific for VAP and included atelectasis in 16.3% of patients, acute pulmonary edema in 11.8%, and ARDS in 6.5%. Using electronic records to identify complications related to ventilation is easy and identifies sick patients, but many patients with VAP were not identified in both studies and therefore VACs and IVACs may be different diseases with different pathobiological causes than VAP.

Sinuff and associates [17] studied the impact of a 2-year multi-faceted intervention via educational sessions supplemented with reminders and led by local opinion leaders on improving concordance with VAP prevention and treatment guidelines and assessed sustainable behavior changes in the ICU. Over time, there was more improvement in prevention strategies than in therapy approaches, and, overall, a significant increase in guideline concordance (aggregate concordance (mean (standard deviation)): 50.7% (6.1), 54.4% (7.1), 56.2% (5.9), 58.7% (6.7); *P* = 0.007). They also observed a reduction in VAP rates (events/330 patients: 47 (14.2%), 34 (10.3%), 38 (11.5%), 29 (8.8%); *P* = 0.03) over the study period, but ICU mortality and length of ICU stay were unchanged, despite adjustments for age and SOFA score. The best concordance rate achieved was only 58.7% and highlights the potential barriers to guideline implementation and variable practices that exist within the community despite multiple reinforcements.

In another study, including 350 patients, investigators using data from electronic medical records compared the incidence and outcomes in VAP patients using various definitions, including the new CDC ventilator-associated event algorithm, before and after a VAP bundle was introduced in their institution (pre-bundle period January 2003 to December 2006 (n = 213); post-bundle period January 2007 to December 2009 (n = 137)) [18]. Unlike the previous study, VAP and VAC incidence remained unchanged and was not affected by the implementation of the VAP bundle despite good compliance. However, mortality adjusted for severity of illness was less in the post-bundle period (23% versus 18%, *P* <0.0001), although the duration of MV, ICU and hospital LOS did not change post-bundle introduction. The lack of reduction in VAP and VAC incidence could have been due to continuous quality improvement interventions that were already underway prior to guideline implementation, but interestingly the newer ventilator-associated event definitions did not recognize VAP in all patients, similar to findings from the studies by Muscedere and colleagues [15] and Hayashi and colleagues [16] discussed above.

Luna and associates [19], in a prospective study of 283 ventilated patients, analyzed if an antibiotic prescription strategy based on routine endotracheal aspirate (ETA) culture was better than empiric antibiotic therapy for VAP, as outlined by the American Thoracic Society (ATS)/Infectious Disease Society of America (IDSA) guidelines. Eighty-three patients had VAP and the ETA and bronchoalveolar lavage (BAL) cultures had concordance in only 52 culture pairs. Sensitivity for ETA to predict a BAL-obtained pathogen was 62.4% (78/125 microorganisms cultured) and was better if done within 3 days of VAP onset and in recurrent VAP. Antibiotic decisions made according to the ATS/IDSA guidelines led to appropriate therapy in 97.9% of patients compared with 77.4% based on ETA culture, with fewer antibiotic days using the ETA-based culture. Hence, using a strategy for VAP diagnosis and treatment decision-making based on ETA cultures alone could result in inappropriate therapy but possibly helps with de-escalation and leads to fewer antibiotic days.

## Community-acquired pneumonia and healthcare-associated pneumonia

Recent studies have confirmed the significant heterogeneity among HCAP patients and also that the risk for MDR pathogens has regional differences. In a study including 519 patients with CAP and 419 with HCAP, the authors compared the performance of Pneumonia Severity Index (PSI) and CURB-65 risk scores for predicting 30-day mortality [20]. HCAP patients were sicker, had more frequent ICU admission, longer length of ICU stay and higher mortality than CAP patients in this cohort. The discriminatory power for 30-day mortality, using both PSI and CURB-65, was lower in HCAP patients than CAP patients (AUC for PSI = 0.679, CURB-65 = 0.599 in HCAP group versus AUC for PSI = 0.835, CURB 65 = 0.79; *P* = 0.009). Thus, both scoring systems were less effective for predicting mortality in HCAP than in CAP patients, but if used, the PSI scoring system performed better than CURB-65. In a prospective study including 1,413 patients (887 CAP and 526 HCAP), Shindo and associates [21] determined the risk factors for pathogens resistant to macrolides, beta-lactams and respiratory fluoroquinolones (CAP-DRPs). HCAP patients had a higher frequency of CAP-DRPs than CAP patients (26.6% versus 8.6%) and a higher 30-day mortality rate (20.3% versus 7.0%). Independent risk factors for CAP-DRPs were similar in both CAP and HCAP groups, and included prior hospitalization, immunosuppression, previous antibiotic use, gastric acid-suppressive agents, tube feeding, and non-ambulatory status. The higher the number of risk factors, the greater the chance of CAP-DRPs (AUC 0.79, 95% CI 0.74 to 0.84). They also identified risk factors for methicillin-resistant *Staphylococcus aureus* (MRSA), which included dialysis within 30 days, prior MRSA isolation within the past 90 days, antibiotics in the past 90 days, and gastric acid-suppressive therapy. However, the presence of a high frequency of resistant pathogens in this study group limits generalization and further studies are needed for external validity of the model.

In another study, Aliberti and colleagues [22] used probabilistic risk scores for prediction of MDR pathogens in two independent cohorts admitted to the hospital from the community (n = 3,474) to validate the previously reported Shorr and Aliberti risk scores. The prevalence of MDR pathogens was 7.6% in Barcelona and 3.3% in Edinburgh and the two scores performed consistently better than the traditional HCAP classification in both centers. Maruyama and colleagues [23], in a prospective study of 425 patients (CAP = 124, HCAP = 321), applied a therapeutic algorithm based on the presence of MDR risk factors (immunosuppression, hospitalization within the last 90 days, poor functional status indicated by a Barthel Index score <50, and antibiotic therapy within the past 6 months) and severity of illness (need for ICU admission or requiring MV) to determine its impact on outcomes. HCAP patients with no or one risk factor were treated with CAP therapy and those with two or more risk factors were treated with a hospital acquired pneumonia regimen based on the ATS/IDSA 2005 guidelines. HCAP patients with two or more risk factors had a higher incidence of MDR pathogens and higher mortality than CAP patients (27.1% versus 2%, *P* <0.001, and 13.7% versus 5.6%, *P* = 0.017, respectively). Although only 53% of HCAP patients received broad-spectrum antibiotics, using the algorithm the majority (92.9%) received appropriate therapy for the identified pathogens. Thus, using this approach, broad-spectrum antibiotic use can be limited, even in patients with HCAP. Lacroix and associates [24] investigated the role of early fiberoptic bronchoscope-guided distal protected small volume bronchoalveolar lavage (mini-BAL) in 54 HCAP patients. Mini-BAL helped identify causative pathogens more efficiently than blood culture (46.3% versus 11.1%, *P* <0.01), up to 72% in patients who did not receive prior antibiotics. Thus, a strategy based on mini-BAL might help with early identification, but the authors did not compare length of antibiotic days, development of resistance or mortality between an empiric regimen and patients who had mini-BAL. The practicality of this approach in the non-intubated HCAP population needs to be validated.

Sicot and colleagues [25] evaluated the characteristics of 161 patients with Panton-Valentine leucocidin (PVL) community-acquired *S. aureus* pneumonia from a French registry, based on methicillin resistance. Both PVL-MRSA (n = 37, 23%) and PVL-methicillin-sensitive *Staphylococcus aureus* (PVL-MSSA; n = 124, 77%) occurred in younger patients (median age 22.5 years) with no underlying comorbidities. Airway hemorrhage was more frequent in PVL-MSSA necrotizing pneumonia compared with PVL-MRSA (44.2% versus 24.1%, *P* = 0.056) but there was no significant difference in mortality (39.4% versus 37.9%), ICU admission, severity of disease or use of antibiotics between the two groups. Interestingly, methicillin resistance was not associated with increased mortality, but patients with airway hemorrhage had a three-fold increase in 7- and 30-day mortality (OR 3.75 and 3.68, respectively) and patients treated with an anti-toxin regimen (clindamycin, linezolid, or rifampicin) had a better chance of survival (mortality rate 6.1% versus 52.3%, *P* <0.001) even though the timing of therapy was not available. This study is one of the largest series on necrotizing community-acquired staphylococcal infection and shows that, despite the resistance pattern, PVL-associated *S. aureus* infection can be a severe disease with high mortality in young patients from the community and that the use of anti-toxin therapy in suspected patients is associated with a potential survival advantage.

## Viral infection in the critically ill patient

Choi and associates [26] studied the role of viruses in 198 patients with severe pneumonia (64 with CAP and 134 with HCAP) using RT-PCR and BAL fluid (58.1%) or nasopharyngeal swab (84.1%). Of the patients, 35.9% (n = 71) had positive bacterial culture, 36.4% (n = 72) had viral infections, and 9.1% (n = 18) had bacterial-viral co-infections. Rhinovirus was the most commonly identified virus (23.6%), followed by parainfluenza virus (20.8%) and human metapneumovirus (18.1%). Bacterial co-infection was more common with parainfluenza and influenza viruses and less common with respiratory syncitial virus and rhinoviruses. There was no difference in mortality between each group, but of those patients with viral infection, rhinovirus was associated with the highest mortality (52.9%), followed by influenza virus (33.3%). This is an interesting study and shows that polymicrobial infection with viruses and bacteria is not uncommon in patients with severe pneumonia. However, some study participants had antibiotics prior to BAL and therefore the negative bacterial cultures may not have been an accurate finding.

In contrast to the discussion above, bacterial infection commonly complicates viral respiratory infection and is often associated with higher morbidity and mortality. Muscedere and colleagues [27] evaluated the risk of coexistent or secondarily acquired bacterial respiratory tract or bloodstream-positive cultures in 681 patients with influenza A (H1N1) infection during the 2009 outbreak. They noted that 38% of patients (n = 259) had at least a positive blood or respiratory culture during their ICU stay (29.7% had co-existent and 44.4% had ICU-acquired infection; 15.4% had both) despite almost all patients receiving antibiotics. Patients with any positive culture had higher morbidity with more days on the ventilator, longer ICU and hospital LOS and higher hospital mortality (24.7% versus 19.9%, *P* = 0.15). The interesting finding from this study is that influenza infection (H1N1) is not as mild as previously thought; the majority of ICU patients required MV and the morbidity and mortality were high even in patients without bacterial co-infection. Hung and colleagues [28] in a double-blind, randomized controlled trial evaluated the use of hyperimmune IV immunoglobulin (H-IVIG) fractionated from convalescent plasma of patients who had 2009 H1N1 infection (n = 17) versus normal IV immunoglobulin (n = 18) in 35 patients with severe H1N1 infection. Patients who received H-IVIG had significantly lower viral loads post-treatment and, if treatment was given within 5 days of onset, had mortality benefit (OR 0.14, 95% CI 0.02 to 0.92, *P* = 0.04). Although the study is limited by a relatively small sample size, the H1N1 antibody present in the convalescent H-IVIG, if used early, offers potential benefit in the treatment of H1N1 infection.

## Nosocomial pneumonia

The ATS/IDSA guidelines recommend antibiotic therapy based on the risk for MDR pathogens with early onset infection (within 5 days of admission), generally using a narrow-spectrum antibiotic regimen. Restrepo and colleagues [29] examined the microbial cultures of 496 VAP patients from 2 large prospective, randomized, open-label studies, classifying patients as early- (<5 days since hospitalization, n = 248) and late-onset VAP (>5 days, n = 248). Late-onset VAP patients had a higher overall frequency of Gram-negative pathogens (84.3% versus 75.4%, *P* = 0.02) and more significant antibiotic exposure in the prior month (85.5% versus 68.5%, *P* <0.01). However, both early- and late-onset VAP patients had similar rates of MDR pathogens (27.8% and 32.3%, respectively, *P* = 0.33). Investigators from the EU-VAP study divided 485 patients with microbiology-confirmed nosocomial pneumonia into two groups; group 1 was early-onset with no MDR risk factors (n = 152) and group 2 was early-onset with MDR risk factors or late-onset pneumonia [30]. The presence of severe sepsis/septic shock (OR = 3.7) and pneumonia that developed in a center with greater than a 25% prevalence of resistant pathogens (OR = 11.3) was independently associated with the presence of resistant pathogens in group 1 patients. These findings suggest that most patients with VAP are at risk for MDR pathogens, and that very few can safely receive narrow-spectrum empiric therapy.

Tumbarello and associates [31] analyzed the impact of multi-drug resistance on outcomes in 110 patients admitted to ICU with culture-confirmed PA pneumonia. Forty-two cases (38%) involved MDR PA, and 9 (8.1%) were colistin-only susceptible PA. The initial antimicrobial regimen was inadequate in 56 patients (50.9%) and more often inadequate among those with MDR PA. Patients who had initial inappropriate antibiotics had a higher mortality than those who had appropriate therapy (64.2% versus 24.7%, *P* = 0.001) and MDR PA patients treated with empiric combination therapy had a lower risk of initial inappropriate antibiotics than those treated with monotherapy. In a similar study, Pena and colleagues [32] looked at the impact of MDR in 91 patients with PA VAP, of which 60 cases were caused by MDR strains, 42 (70%) of which were extensively drug-resistant. As with the previous study, VAP patients with susceptible PA received adequate empiric antibiotic coverage more often, both empiric and definitive, than patients with MDR pathogens (68% versus 30%, *P* <0.001). Although inadequate antibiotics were an independent risk factor for early mortality (OR 4.27, *P* = 0.052) and patients with susceptible strains had more adequate coverage, those with inadequate therapy had a higher mortality that could be related to severity of illness more than to resistance. The OUTCOMEREA data on PA pneumonias include 393 PA-VAP episodes with multi-drug resistance defined as resistance to two antibiotics (piperacillin, ceftazidime, imipenem, colistin, and fluoroquinolones) [33]. MDR was not related to treatment failure or relapses but was associated with longer ICU LOS. Fluoroquinolone use prior to the first episode was associated with increased risk of treatment failure probably related to the induction of resistance, but when used in the treatment regimen, fluoroquinolones decreased the risk of treatment failure. In another study of 143 confirmed pseudomonal pneumonia patients, serotypes O6 and O11 were more prevalent, but mortality was higher with O1 (40%) and lower with O2 (0%); clinical resolution tended to be better with O2 (82%) compared with other serotypes. Higher Acute Physiology and Chronic Health Evaluation II score was associated with worse outcomes among all serotypes [34].

Clinically feasible and simple to use predictors of ICU outcome in patients with ICU-acquired pneumonia are important in clinical practice. In a prospective observational study, Esperatti and colleagues [35] determined the usefulness of a set of predictors of adverse outcomes (PAOs) in 355 ICU-acquired pneumonia patients and determined their correlation with serum inflammatory markers and clinical prognostic scores. The PAOs were determined 72 to 96 hours after starting antibiotics (evolutionary criteria), and were considered positive if there was: 1) no improvement in partial pressure of oxygen in arterial blood/fraction of inspired oxygen ratio since the onset of pneumonia and in the absence of other causes of worsening oxygenation; 2) requirement for intubation despite antibiotics for 24 hours; 3) persistence of fever or hypothermia together with purulent secretions; 4) a 50% or greater increase in pulmonary infiltrates on chest radiograph; 5) development of septic shock or multi-organ dysfunction not present on day 1. Fifty percent of patients had at least one PAO, and had a higher 28-day mortality (45% versus 19%, *P* = 0.001), less mean ventilator-free days (10 versus 12, *P* = 0.001) and elevated serum inflammatory markers such as PCT and C-reactive protein compared with those who did not have any PAOs. The trend remained significant in patients who developed VAP, as well as those who had non-ventilator ICU-acquired pneumonia. The failure to improve oxygenation (partial pressure of oxygen in arterial blood/fraction of inspired oxygen) and a worsening SOFA score over 5 days were independently associated with mortality in a multivariate analysis.

## New insights into treatment strategies

The 2007 ATS/IDSA guidelines recommend using combination antibiotic therapy in patients with severe CAP admitted to the ICU. Adrie and colleagues [36] examined the impact of dual (β-lactam plus macrolide or fluoroquinolone (n = 394)) versus monotherapy (β-lactam alone (n = 471)) in immunocompetent severe CAP patients, using a large prospective database. They found no significant difference in 60-day mortality between patients who had dual therapy compared to monotherapy, and in those who received dual therapy, there was no survival advantage between the macrolide and fluoroquinolone subgroups (subdistribution hazard ratio 1.45, 95% CI 0.78 to 2.70, *P* = 0.24). Interestingly, patients who had initial adequate antibiotic therapy had a survival advantage (subdistribution hazard ratio 0.63, 95% CI 0.42 to 0.94.00, *P* = 0.02) and those who received dual therapy had a higher frequency of initial adequate antibiotics, which did not translate into improved survival. Further, subgroup analysis did not reveal a survival benefit even in patients with septic shock or *Streptococcus pneumoniae* infection receiving dual therapy, but dual therapy did not increase the development of MDR pathogens or nosocomial pneumonia. In a similar study including 3,203 hospitalized patients, guideline concordant therapy (defined as macrolides/β-lactams or respiratory fluoroquinolone monotherapy) did not have a mortality benefit compared with discordant therapy, but a composite endpoint of death or ICU admission was lower in the concordant group (14.7% versus 29.0%; adjusted OR 0.44, 95% CI 0.36 to 0.54, *P* <0.0001) [37]. Most patients received levofloxacin monotherapy in the guideline-concordant group (70%) and there was no significant difference in mortality between patients who received macrolide/β-lactam antibiotics versus those who had fluoroquinolone/β-lactams (adjusted OR 0.75, 95% CI 0.395 to 1.42, *P* = 0.38). The findings from these studies contradict previous reported studies, but lack of randomization and possible misclassification bias limits interpretation.

Antibiotic dosing in critically ill patients is challenging due to deranged drug metabolism and elimination that can lead to suboptimal dosing. Extended infusion of antibiotics with a time-dependent killing mechanism, such as beta-lactams, has been proposed as a means to overcome the pharmacokinetic/pharmacodynamic (PK/PD) alterations in severely ill patients in order to optimize the time that drug concentration exceeds the minimum inhibitory concentration (MIC) of the target organism. Carlier and colleagues [38] studied the effect of augmented renal clearance on extended infusion of meropenem or piperacillin/tazobactam (Pip/Tazo) in 61 patients with sepsis and normal creatinine clearance [38]. Patients received a loading dose (1 g for meropenem and 4.5 g for Pip/Tazo) followed by extended infusion usually over 3 hours every 6 hours for Pip/Tazo and 8 hours for meropenem. Only 55% of patients achieved a predefined PK/PD target, and of patients who had augmented renal clearance (48%), the majority did not achieve the target (76%). Augmented renal clearance with a clearance >130 ml/minute was an independent predictor of not achieving the PK/PD target, but the study was not designed to look at outcome and treatment failures, and the PK/PD target may have been set too high. Dulhunty and colleagues [39], in a double-blind randomized controlled trial, compared continuous versus intermittent bolus dosing of Pip/Tazo, meropenem, and ticarcillin-clavulanate in 60 patients with severe sepsis. Patients in the intervention arm received active infusion and placebo boluses and controls received placebo infusion and active boluses. The concentration exceeded the MIC more often in the intervention group than in controls (81.8% versus 28.6%, *P* = 0.001; most with meropenem and least with ticarcillin-claculanate) and the patients in the intervention group had a higher clinical cure rate, but there was no difference in ICU or hospital LOS or mortality. The study reinforces the dosing options available for critically ill patients based on PK characteristics, but did not have the statistical power to determine a mortality benefit, although there was a trend towards better survival in the intervention arm.

With the growing development of resistance to beta-lactams, aminoglycosides are advocated for patients with severe sepsis as part of combination therapy, especially with PA infection and the bactericidal activity of aminoglycosides is dependent on peak concentration (Cpeak) relative to MIC. As noted above, the concentration of aminoglycoside can change in critically ill patients due to variations in drug clearance. In a study of 63 patients with severe sepsis (50% with lung infection) requiring amikacin, investigators used therapeutic drug monitoring and dose adjustments to optimize serum concentration [40]. Microbiological eradication and clinical cure were higher in patients who achieved initial optimal Cpeak/MIC and were proportionately higher with higher target concentration. Patients who achieved the target concentration after 3 days had a worse clinical cure and microbiological eradication than those who achieved this goal on the first day. Renal failure was seen in 24% of patients and was more likely in those with impaired clearance and higher minimum concentration.

Inhalation antibiotics have the potential advantage of achieving high alveolar concentrations with minimal systemic side effects. In a matched 1:1 case control study, Tumbarello and colleagues [41] studied aerosolized colistin (given via jet nebulizer or ultrasonic nebulizer) as an adjunctive treatment to intravenous therapy with the same drug in 208 VAP patients with positive cultures for Gram-negative MDR pathogens susceptible only to colistin. Patients receiving aerosolized therapy in conjunction with intravenous colistin had a higher clinical cure rate compared with controls (69.2% versus 54.8%, *P* = 0.03) and fewer days on the ventilator after onset of VAP (8 versus 12 days, *P* = 0.001), but no difference in overall mortality or ICU LOS. Also, there was no difference in the rate of new-onset kidney failure between the two groups. The study results are in contrast to previous reports with aerosolized colistin providing only modest benefits. In this study the medication was delivered in the majority of patients using conventional ventilators with jet nebulizers and the local concentration of antibiotics could not be determined. In view of the reported increased incidence of drug-resistant pathogens causing VAP and the potential treatment alternative with aerosolized colistin, further randomized controlled studies are needed prior to generalization of the results.

In a study looking at factors influencing antibiotic de-escalation in 229 patients admitted to ICU with sepsis, only 51.1% of patients had the number of antibiotics reduced or switched to a narrower spectrum [42]. However, there was no difference in mortality rate, ICU LOS or duration of MV between patients who had de-escalation compared with those with no de-escalation. In those patients who did not have de-escalation, 15% had no de-escalation despite meeting criteria. Narrow-spectrum initial antibiotic therapy (OR 0.1, 95% CI 0.0 to 0.1, *P* <0.001) and infection with an MDR bacteria (OR 0.2, 95% CI 0.1 to 0.7, *P* = 0.006) were factors preventing de-escalation.

Duration of antibiotic treatment for nosocomial pneumonia is not clearly defined, and previous studies have shown that a short duration may be as clinically effective as a longer duration (>14 days) and more cost-effective. A meta-analysis of four randomized controlled trials (including 883 patients) comparing short (7 to 8 days) with long (10 to 15 days) duration regimens in patients with VAP showed no difference in mortality, ICU LOS or MV between the two groups, and more antibiotic-free days in the short course group [43]. There was a trend towards more relapses due to non-fermenting Gram-negative bacilli in the shorter duration antibiotic cohort. In another observational study, including 89 suspected VAP patients with negative BAL results, investigators compared the effects of early discontinuation (antibiotics stopped within 1 day of final negative quantitative BAL culture results) with late discontinuation of antibiotics (more than 1 day after negative final BAL cultures) [44]. There was no difference in mortality between early discontinuation (25.0%) and late discontinuation (30.6%) patients (*P* = 0.642). Clinical resolution as noted by Clinical Pulmonary Infection Score was similar in both groups and patients with late discontinuation had a longer duration of antibiotic therapy (9 versus 4 days, *P* <0.001). Interestingly, patients with early discontinuation developed less frequent superinfections compared with late discontinuation patients (22.5% versus 42.9%, *P* = 0.008). These results add credence to the value of de-escalation for VAP patients and to the possibility that longer antibiotic courses may cause microbial persistence and selection pressure leading to the development of microbial resistance.

## Prevention

Prophylactic systemic antibiotics have a role in preventing early-onset VAP in closed head injury patients. Valles and colleagues [45] evaluated the role of single-dose antibiotics within 4 hours of intubation (ceftriaxone 2 g intravenously; 1 g ertapenem in those with hypersensitivity to beta-lactam; 500 mg levofloxacin in those with anaphylaxis to beta-lactam) in the prevention of early-onset VAP or ventilator-associated tracheobronchitis in comatose patients. They compared 71 patients who received prophylaxis to 58 historical cohorts. The patients who received prophylaxis had fewer microbiologically confirmed cases of VAP (7% versus 27.6%, OR 0.11, *P* = 0.009), less MV days, and shorter ICU LOS. However, there was no difference in mortality or hospital LOS between the two groups. Although there was no increased incidence of MDR pathogens in the prophylaxis group with late-onset VAP, the study patients did not have surveillance cultures and hence the rate of colonization is unknown. Prophylactic antibiotic at the time of intubation in high-risk patients at risk for VAP is an interesting concept and further prospective randomized controlled studies are required prior to generalization of the results.

Statins have possible anti-inflammatory and immunomodulatory effects and their use in patients with pneumonia had previously been reported to lead to beneficial outcomes. Papazian and associates in a double-blind, parallel-group study, randomized VAP patients (defined as having a Clinical Pulmonary Infection Score >5) to receive simvastatin (60 mg) or placebo [46]. The authors planned to enroll 1,002 patients, but the study was stopped prematurely because of futility after enrolling 153 in the intervention arm and 147 in the control group. There was no significant difference in 28-day mortality (6% absolute increase with simvastatin) or other secondary outcomes, including duration of MV, coronary events, ARDS, or adverse side effects between the two groups. However, of those patients naive to prior statin use, the 28-day mortality was higher in the placebo arm (28% versus 5%, *P* = 0.01). Although this trial was underpowered to highlight any marginal beneficial effects of statins, the results are similar to another recent trial exploring the role of statins in sepsis that also did not find any difference in levels of interleukin-6, but possible beneficial effects in continuing chronic statin therapy [47].

Probiotics may restore non-pathogenic gut flora and the value of their use in critically ill patients has been inconclusive. Barraud and associates [48] conducted a meta-analysis including 13 randomized studies with 1,439 patients to evaluate the use of probiotics (most with *Lactobacillus* sp.) in the ICU. Probiotic use did not have a significant impact on mortality or the duration of MV. However, probiotic use resulted in a significant decrease in nosocomial pneumonia even after adjustment for heterogeneity (OR 0.54, 95% CI 0.36 to 0.79) and also led to a shorter ICU LOS. Use of probiotics could potentially prevent gastric colonization by pathogenic bacteria and might explain the beneficial effects seen with ICU-acquired pneumonia. Whether this should be added to VAP prevention measures is still to be determined and will need further large trials, with VAP as the primary end point.

## Conclusion

Respiratory infections remain the most common cause of sepsis and septic shock, with Gram-negatives being slightly more common than Gram-positives. Some patients have sepsis with negative cultures and these patients may have a better prognosis than those with positive cultures. Severe infection is part of a multiple system illness, and some recent data have examined the relationship of pneumonia to cognitive impairment, showing that infection can lead to cognitive decline, possibly related to inflammatory cytokines, while at the same time patients who develop pneumonia may be more cognitively impaired than those without pneumonia. Many episodes of pneumonia result from gastric aspiration, but recent investigations have shown that development of VAP could not be prevented even with enteral feeding and close attention to gastric residual volume. One alternative is to place feeding tubes directly into the small bowel, which may reduce pneumonia risk but not have an impact on mortality.

Biomarkers may help us guide the need for therapy, the duration of therapy for pneumonia, and the prognosis for survival, but most data have been collected with serum PCT measurements. Recent studies suggest that kallistatin is a protective hormokine, and that, in the setting of septic shock, low levels may predict adverse outcomes such as ARDS and death. Another biomarker, presepsin, is elevated in severe sepsis patients who die. PCT has been used to separate patients with infection from those without infection, but in the presence of CHF new data suggest that low levels may rule out infection, but that severe heart failure itself can falsely elevate levels.

The diagnosis of VAP remains confusing, and new data have shown the limited value of the CDC definition of VACs. VACs include a number of non-infectious diagnoses, and many patients with VAP do not have a VAC. Some data suggest that VACs can be prevented, but there are also studies showing that the currently available ventilator bundles cannot prevent them. In the management of CAP and VAP, it is important to account for MDR pathogens in empiric therapy. Outside the hospital, there are patients who develop HCAP and many of these are also at risk for MDR pathogens. New studies have suggested strategies to identify patients at risk for resistant pathogen infection, and therapies that optimize efficacy, without the overuse of broad-spectrum therapy. The optimal therapy of MDR pathogens is being explored, but for MRSA CAP, the use of anti-toxin therapy may improve outcome. In VAP, the role of resistance in determining outcome is uncertain, but most studies suggest an interaction between drug susceptibility and disease severity. Optimizing the therapy of MDR pathogens is being explored in a number of ways, including the use of modified dosing regimens, and inhaled antibiotics for pneumonia. Our enhanced understanding of altered renal clearance in severe infection has led to renewed efforts to provide enough antibiotic to seriously ill patients, and to avoid the use of too low a dose of an effective agent.

In the future, we will continue our efforts at pneumonia prevention, but this will require a continued understanding of disease pathogenesis, the use of prevention bundles and the application of standard therapies in novel ways (as demonstrated with studies of statins).

## Note

This article is part of a collection of Year in review articles in *Critical Care*. Other articles in this series can be found at [49].

## Abbreviations

ARDS: Acute respiratory distress syndrome
ATS: American Thoracic Society
AUC: Area under the curve
BAL: Bronchoalveolar lavage
CAP: Community-acquired pneumonia
CDC: Centers for Disease Control and Prevention
CHF: Congestive heart failure
CI: Confidence interval
Cpeak: Peak concentration
ETA: Endotracheal aspirate
GRV: Gastric residual volume
HCAP: Healthcare-associated pneumonia
H-IVIG: Hyperimmune IV immunoglobulin
IDSA: Infectious Disease Society of America
IVAC: Infection-related ventilator-associated complication
LOS: Length of stay
MDR: Multi-drug resistant
MIC: Minimum inhibitory concentration
MRSA: Methicillin-resistant *Staphylococcus aureus* MSSA, Methicillin-sensitive *Staphylococcus aureus*
MV: Mechanical ventilation
OFA: Sequential Organ Failure Assessment
OR: Odds ratio
PA: *Pseudomonas aeruginosa*
PAO: Predictors of adverse outcome
PCR: Polymerase chain reaction
PCT: Procalcitonin
Pip/Tazo: Piperacillin/tazobactam
PK/PD: Pharmacokinetic/pharmacodynamic
PSI: Pneumonia Severity Index
PVL: Panton-Valentine leucocidin
RT: Reverse transcriptase
VAC: Ventilator-associated complication
VAP: Ventilator-associated pneumonia
